# Repeat Syphilis Infection and HIV Coinfection Among Men Who Have Sex With Men — Baltimore, Maryland, 2010–2011

**Published:** 2013-08-16

**Authors:** Patrick Chaulk, Ravikiran Muvva, James Morgan, Marcia L. Pearl, Sandra Matus, David Blythe, Colin Flynn, Alexandra M. Oster, Gabriela Paz-Bailey, Phyllis Burnett, Glen Olthoff, Geoffrey Hart-Cooper, Laura A. Cooley, Christine Ross

**Affiliations:** Baltimore City Health Dept; Baltimore County Dept of Health and Human Svcs; Maryland Dept of Health and Mental Hygiene; Div of HIV/AIDS Prevention; Div of STD Prevention, National Center for HIV/AIDS, Viral Hepatitis, STD, and TB Prevention; CDC Experience Applied Epidemiology Fellowship; EIS officers, CDC

Syphilis diagnoses in the United States have increased substantially over the past decade, and most cases occurred among men who have sex with men (MSM). Nationally, rates of primary and secondary (P&S) syphilis reported among men increased, from 3.0 cases per 100,000 population in 2001 to 8.2 in 2011 (1). In 2011, approximately 72% of P&S syphilis cases occurred among MSM* (1), among whom new diagnoses of human immunodeficiency virus (HIV) infection have increased in recent years (2). Infection with syphilis increases the likelihood of acquiring and transmitting HIV; moreover, the occurrence of syphilis in an HIV-infected person is an indication of behavior that might increase the likelihood of HIV transmission (3). The population of Baltimore, Maryland, is particularly affected by syphilis and HIV. In 2011, the Baltimore metropolitan statistical area (MSA) had the second highest rate of reported cases of P&S syphilis (11.4 per 100,000 population) (1) and the sixth highest estimated rate of diagnoses of HIV infection (33.8 per 100,000 population) (2) compared with other MSAs in the United States. Local public health officials have noted a subpopulation of MSM diagnosed with repeat syphilis infection; they believe that this subpopulation might bear a disproportionate burden of both syphilis and HIV infection and that intensifying syphilis and HIV prevention efforts among this subpopulation might reduce syphilis and HIV transmission overall in the Baltimore area.

The Maryland Department of Health and Mental Hygiene requested assistance from CDC to describe this subpopulation and identify characteristics that could be used to improve the selection and delivery of syphilis and HIV prevention interventions. CDC, the Maryland Department of Health and Mental Hygiene, the Baltimore City Health Department, and the Baltimore County Department of Health and Human Services analyzed data from sexually transmitted disease and HIV surveillance and from interviews conducted by health department staff members for the purpose of contact tracing during 2007–2011. MSM (as determined by risk behaviors reported in surveillance and interview records) aged ≥15 years who resided in Baltimore city or Baltimore County and were diagnosed with repeat syphilis infection were included in this analysis. Persons were considered to have repeat syphilis infection if they were reported to have early (primary, secondary, or early latent) syphilis diagnosed in 2010 or 2011 and had received treatment for a previous syphilis diagnosis during 2007–2011, as documented in electronic sexually transmitted disease surveillance records.

In all, 493 early syphilis cases in 2010 and 2011 were reported among 460 MSM; the number of diagnoses increased 29%, from 215 in 2010 to 278 in 2011. Of these 460 MSM, 92 (20%) were determined to have repeat syphilis infection; 77 of these 92 MSM (84%) had two syphilis diagnoses during 2007–2011, and 15 MSM (16%) had three or more syphilis diagnoses during that period. Median time between the two most recent syphilis diagnoses was approximately 18 months; 26% occurred ≤12 months apart. For the most recent syphilis diagnoses, only 5% were primary syphilis, whereas 41% were secondary syphilis, and 53% were early latent syphilis. Median age was 30.5 years (range: 19–62 years), 83 patients (90%) were black, and 85 (92%) resided in Baltimore city. Seventy-nine (86%) were diagnosed with HIV before or at the time of their most recent syphilis diagnosis.

Syphilis case reports among MSM increased in Baltimore from 2010 to 2011, and one in five MSM with syphilis had repeat infection. A substantial proportion of repeat syphilis infections occurred ≤12 months apart. Also, very few men were diagnosed with primary syphilis, suggesting possible missed opportunities for early diagnosis and longer periods of infectiousness. The majority of MSM in Baltimore with repeat syphilis infection are living with HIV. Because repeat syphilis infection can be an indicator of continued engagement in behaviors associated with acquisition and transmission of HIV and other sexually transmitted diseases, MSM with repeat syphilis should be prioritized for comprehensive prevention services, including risk reduction counseling, increased access to condoms, and increased frequency of syphilis testing (every 3–6 months), with active outreach for missed testing appointments. Testing, educational, and outreach interventions targeted to preventing future syphilis and HIV infections among MSM with repeat syphilis infection might mitigate the spread of syphilis and HIV among MSM overall in Baltimore.

## Footnotes

* Among cases in the 47 jurisdictions that reported the sex of sex partners.

## References

[1] CDC (2012). Sexually transmitted disease surveillance, 2011.

[2] CDC (2013). HIV surveillance report, 2011.

[3] CDC (2009). Guidelines for prevention and treatment of opportunistic infections in HIV-infected adults and adolescents: recommendations from CDC, the National Institutes of Health and the HIV Medicine Association of the Infectious Diseases Society of America. MMWR.

